# Mental Health and Quality of Life of Patients with Differentiated Thyroid Cancer Pre and Post Radioactive Iodine Treatment: A Prospective Study

**DOI:** 10.3390/jcm13185472

**Published:** 2024-09-14

**Authors:** Te-Chang Changchien, Yung-Chieh Yen, Yung-Chuan Lu

**Affiliations:** 1 Department of Psychiatry, E-Da Hospital, Kaohsiung City 824, Taiwan; 2 School of Medicine, I-Shou University, Kaohsiung City 824, Taiwan; 3 Division of Endocrinology and Metabolism, Department of Internal Medicine, E-Da Hospital, Kaohsiung City 824, Taiwan

**Keywords:** differentiated thyroid cancer, health-related quality of life, anxiety, depression

## Abstract

**Background**: Although patients with differentiated thyroid cancer (DTC) have a good prognosis, their long-term clinical course can influence their mental health and their health-related quality of life (HRQoL). However, few studies have evaluated the psychological factors that influence subsequent HRQoL in this population, particularly during the initial treatment stage. **Methods**: In this 1-month cohort study, we evaluated depressive and anxiety symptoms and HRQoL of patients with DTC and examined possible predictors of further HRQoL impairment. **Results**: In total, 181 patients completed questionnaires where they self-rated their psychological status (the Chinese Health Questionnaire [CHQ], Taiwanese Depression Questionnaire [TDQ]) and HRQoL (the 36-item Short Form Health Survey [SF-36]) at baseline and 1 month after radioactive iodine (RAI) therapy. Compared with the general Taiwanese population, patients with DTC reported a worse HRQoL in all dimensions of the SF-36. Multivariate regression models indicated that anxiety and depressive symptoms were inversely correlated with some dimensions (physical functioning, bodily pain, and general health perceptions for the CHQ; role limitations due to physical problems and social functioning for the TDQ). However, psychiatric follow-up and treatment history were significantly associated with physical functioning and role limitations owing to the physical problem dimensions of HRQoL. **Conclusions**: In conclusion, although anxiety and depressive symptoms may negatively affect certain HRQoL domains, psychiatric follow-up can improve the physical dimensions.

## 1. Introduction

The global incidence of thyroid cancer, the most prevalent type of endocrine malignancy, has exhibited a marked rise [1]. Differentiated thyroid cancer (DTC), encompassing the papillary and follicular types, represents over 90% of all thyroid cancers. Thyroid dysfunction is closely associated with psychiatric diseases. It has been demonstrated that thyroid gland malfunctions may result in neuropsychiatric symptoms, particularly affecting cognitive functions and emotional states. In cases of hypothyroidism, the most common neuropsychiatric symptoms include depression, intellectual disability, fatigue, delusions, hallucinations, and even dementia. The aforementioned symptoms, both physical and mental, have the potential to cause distress and negatively impact the quality of life of patients [2]. Depression is a prevalent mental disorder. A review of the literature reveals that between 8 and 17 percent of patients with primary depression have subclinical hypothyroidism, defined as the absence of clinical symptoms of hypothyroidism but the presence of elevated TSH levels. Up to 52 percent of patients with refractory depression also exhibit this phenomenon [3]. Furthermore, clinical guidelines for the treatment of refractory depression include concomitant treatment with levothyroxine as an option. The efficacy of this treatment can be confirmed by clinical performance or cerebral function imaging [4]. Although patients with DTC have a good prognosis, they require long-term follow-up to monitor for possible recurrence. This long-term clinical course requires various possible treatments, such as thyroid surgery, radioactive iodine (RAI) treatment (with or without thyroid hormone withdrawal and recombinant human thyroid-stimulating hormone [rhTSH] use), and thyroid hormone suppression therapy, which can considerably impair mental health and health-related quality of life (HRQoL). Patients with DTC frequently exhibit psychological distress following their diagnosis. The distress experienced may fluctuate throughout the course of cancer treatment, particularly during the initial stages. In a study of thyroid cancer survivors, approximately 40% of the subjects reported a high level of fatigue up to 20 years after diagnosis. The HRQoL and psychological distress of these individuals were found to be significantly associated with fatigue [5]. Moreover, patients undergoing RAI must be housed in radiation isolation wards, which may result in feelings of fear regarding radiation exposure, loneliness, and pessimism, and could potentially have a negative impact on mental health, particularly during the process of thyroid hormone withdrawal. After surgery, patients with DTC who are receiving chronic hormone suppression therapy may experience cognitive impairment and depressive disorders [6]. The precise psychological effects observed across different studies varies. A Korean study documented an increase in anxiety and depression among patients with thyroid cancer [7]. A recent cross-sectional study reported that the prevalence of depression and anxiety increased in DTC survivors and identified some clinicopathological characteristics that predicted impaired emotional well-being [8]. Some cross-sectional and observational cohort studies have indicated that individuals with DTC have a lower HRQoL than the general population [9,10,11,12,13]. Hoftijzer et al. included 153 patients with DTC that were treated with thyroid hormone suppression therapy [14]. They observed that patients had a significantly worse quality of life than controls, and that a longer duration of cure from thyroid cancer, but not thyroid-stimulating hormone (TSH) levels, was associated with improved HRQoL. Nevertheless, few studies have evaluated the psychological factors that influence subsequent HRQoL in patients treated with DTC, particularly during the initial treatment stage (before and after RAI).

In this cohort study, we evaluated the association between depression and anxiety symptoms, and the HRQoL of patients with DTC before and one month after RAI therapy; assessed the correlations between HRQoL and various clinicodemographic variables; and examined the possible predictors of poor post-treatment HRQoL.

## 2. Methods

### 2.1. Participants

This prospective observational study recruited 200 consecutive patients with DTC at E-Da Hospital, Kaohsiung City, Taiwan, between January 2015 and December 2018. All patients aged between 20 and 85 years who had just undergone thyroid surgery without RAI therapy, were receiving continuous levothyroxine treatment, and did not have clinical/laboratory evidence of metastasis were included. We excluded patients with cognitive impairments that prevented them from understanding the purpose of the study or completing the questionnaire. Patients were also excluded if they had any other unstable comorbidities that required intensive medical or surgical treatment and could influence their HRQoL, and if they had substance or drug use problems. A face-to-face interview was conducted to complete a questionnaire that rated the psychological status (symptoms of depression and anxiety) and HRQoL at two time points: while participants were hospitalized for therapeutic RAI and one month later. It was required that patients complete the questionnaire in person and alone; completion of the questionnaire by other individuals was prohibited. Each copy of the questionnaire was examined by a specially trained assistant to evaluate the quality. The data on patient age and sex were obtained from medical records. Furthermore, patients were requested to provide information regarding their marital status (married vs. not married) and their highest level of education achieved (0–6 years, 7–12 years, >12 years) using a written questionnaire. Data on any co-morbidities were derived from medical records. Co-morbidities included hypertension, diabetes, history of psychiatric follow-up with treatment (defined as a regular psychiatric service visit with psychopharmacological treatment or psychosocial intervention), and rhTSH use. Free thyroid hormone (free T4) and thyroid-stimulating hormone (TSH) levels were measured from a blood test at baseline. Participants provided written informed consent. The ethics committee of the hospital approved this study.

### 2.2. Study Instruments

#### 2.2.1. 36-Item Short Form Health Survey (SF-36)

SF-36, originally developed by the Medical Outcomes Study, is a valuable instrument in clinical practice and research [15]. The Taiwanese version of the SF-36 has been validated for HRQoL assessment in the Taiwanese population [16,17]. It measures eight dimensions of health: physical functioning (PF), role limitations due to physical problems (RP), bodily pain (BP), general health perceptions (GH), vitality (VT), social functioning (SF), role limitations due to emotional problems (RE), and mental health (MH). The SF-36 can also be divided into two categories: the Physical Component Summary (PCS) and the Mental Component Summary (MCS). Higher scores indicate a personal perception of better HRQoL.

#### 2.2.2. Chinese Health Questionnaire

The Chinese Health Questionnaire (CHQ) is a 12-question, 2-reverse question, 0–1-point questionnaire used to screen for somatic and psychic anxiety symptoms, social dysfunction, self-confidence, and hope, which are all suggestive of nonpsychotic and anxiety symptoms in common mental disorders (CMDs). CHQ scores range from 0 to 12. Higher total scores indicate more severe anxiety symptoms. The questionnaire was derived from the General Health Questionnaire, with the addition of specially designed and culturally relevant items [18]. In a community study, the questionnaire had a sensitivity and specificity of 70% and 95%, respectively, for minor psychiatric morbidities [19]. A Cronbach’s α of 0.84 and an internal consistency of 0.79 were demonstrated for the CHQ [20]. The cutoff point in the Taiwanese community surveys was >4 points.

#### 2.2.3. Taiwanese Depression Questionnaire

The Taiwanese Depression Questionnaire (TDQ) is an 18-question, 0–3-point questionnaire used to screen for clinical depressive symptoms. This is a culturally specific depression self-rating instrument used in Taiwan that contains items related to somatic symptoms of depression, including “I felt tired and weak”, “I frequently had chest tightness”, and “I felt sick: headache, dizziness, palpitation, or abdominal distress” [21]. The subjects rate each item in terms of certain physical and emotional feelings during the previous week. TDQ scores range from 0 to 54. Higher total scores indicate more severe depressive symptoms. It has a Cronbach’s α of 0.90 and a sensitivity and specificity of 0.89 and 0.92, respectively. The cutoff point for the Taiwanese community population was >18 points.

### 2.3. Statistical Analysis

Descriptive results regarding continuous variables are presented as the mean ± standard deviation (SD), and categorical variables are presented as numbers and percentages. We compared the depressive symptoms of patients with versus without rhTSH use before RAI by using the independent samples *t*-test. The one-sample *t*-test was used to compare the means of each dimension of the baseline HRQoL between our patients and a Taiwanese reference population (age: 45–54 years) [16]. In the longitudinal analyses, all factors at baseline (age, sex, body mass index, history of hypertension, and diabetes; psychiatric follow-up and treatment history; CHQ score; TDQ score; and free T4 and TSH levels) were included in the linear regression models to identify variables that could independently predict psychological symptoms (using the TDQ and CHQ scores as dependent variables) and HRQoL (using the scores of the eight dimensions of the SF-36 as dependent variables) at 1 month after RAI therapy. All analyses were performed using SPSS version 19.0 for Windows (IBM, Armonk, NY, USA). This study was approved by the Institutional Review Board of E-Da Hospital.

## 3. Results

### 3.1. Demographic and Clinical Characteristics

A total of 200 participants were enrolled in this study. Their mean age was 49.4 ± 12.7 years, 148 (74%) were female, and their mean body mass index was 25.8 ± 4.7 kg/m^2^. Furthermore, 26% and 14% of patients had a history of hypertension and diabetes, respectively, and 10% received rhTSH before RAI. The prevalence of clinical depressive disorders and CMDs was 14% and 22%, respectively. Moreover, 22 (11%) patients had a history of psychiatric follow-up and treatment (Table 1). A significant difference in baseline free T4 levels and TDQ scores was noted between the subgroups with and without rhTSH use. Patients who did not use rhTSH (or withdrew thyroid hormones) had higher depression levels (Table 2). With regard to HRQoL, our participants had lower scores in most SF-36 domains than did the Taiwanese reference population, with the exception of the “bodily pain” domain. (Table 3). Of the 200 patients, 181 completed the questionnaire twice and were eligible for the analysis.

### 3.2. Multivariate Linear Regression Models

In the “physical functioning” model, a psychiatric follow-up and treatment history (β = 11.60, *p* < 0.05) was significantly associated with HRQoL, whereas anxiety symptoms were negatively associated with this outcome (β = −2.33, *p* < 0.05; *R*^2^ = 0.347). In the model of “role limitations due to physical problems”, a psychiatric follow-up and treatment history was found to be significantly associated with HRQoL, whereas depressive symptoms were negatively associated with it (β = 33.21, *p* < 0.05 and β = −2.82, *p* < 0.001, respectively, *R*^2^ = 0.306). In the “bodily pain” and the “general health perceptions” models, there was an inverse correlation between anxiety symptoms and both bodily pain (β = −3.54, *p* < 0.01) and general health perceptions (β = −3.69, *p* < 0.01; *R*^2^ = 0.3 and 0.323, respectively). In the “social functioning” model, lower levels of depression were identified as a significant predictor of higher levels of social functioning (β = −1.20, *p* < 0.01; *R*^2^ = 0.342). In the CHQ model, female sex was a significant predictor of anxiety symptoms (β = 0.90, *p* < 0.05; *R*^2^ = 0.433). In the TDQ model, a psychiatric follow-up and treatment history had a significant effect on depressive symptoms (β = 5.64, *p* < 0.01; *R*^2^ = 0.412; Table 4). In the remaining models (VT, RE, and MH), no significant variables were identified after adjusting for demographic data, clinical history, and free T4 and TSH levels.

## 4. Discussion

In this study, our main findings were that in the cross-sectional comparison of baseline HRQoL scores in patients with DTC and HRQoL scores in the Taiwanese general population, patients reported a worse HRQoL in all domains of the SF-36. Notably, our longitudinal data indicated that psychiatric follow-up and treatment history were significantly associated with the “physical functioning” and “role limitations due to physical problems” dimensions of HRQoL. This suggests that medical history (regular psychiatric service visits with treatment) is an independent predictor of better physical activity and job (or family) roles. Anxiety (measured by the CHQ score) and depressive symptoms (measured by the TDQ score) were inversely correlated with some dimensions (physical functioning, bodily pain, general health perceptions for the CHQ; role limitations due to physical problems and social functioning for the TDQ). Thus, anxiety symptoms can predict poor daily physical activity, pain control, and self-perception (all physical component scores), whereas depressive symptoms can predict impaired physical roles and social activities.

Few longitudinal studies have focused on the changes in the HRQoL of patients with DTC, even with regard to mental health. No clear evidence exists regarding the association between mental health and HRQoL, and the psychological factors affecting long-term QoL remain unclear. To the best of our knowledge, our longitudinal study is the first to investigate the relationship between psychological symptoms and HRQoL, and to present some remarkable findings after adjusting for common demographic and clinical data. Gou et al. followed 186 patients with DTC for 2 years and reported that this patient group required more attention and longer-term observation than the other patient groups because of lower preoperative and postoperative HRQoL [22]. They also performed univariate and multivariate analyses to predict HRQoL impairment using demographic characteristics and clinical history; however, unlike our study, no psychological variables (such as anxiety and depressive symptoms) were included or controlled for. Gamper et al. reported the HRQoL of 284 patients with DTC compared with the Austrian general population, investigating a disease course of up to 30 months and identifying patient characteristics associated with HRQoL [23]. The persistent detrimental impact on HRQoL (especially in terms of fatigue and role functioning) was unrelated to favorable clinical outcomes. However, the study did not clarify the possible association between mental health and long-term HRQoL.

The World Health Organization defines quality of life as “an individual’s perception of their position in life in the context of the culture and value systems in which they live and in relation to their goals, expectations, standards, and concerns” [24]. “Health-related quality of life” is an individual’s perceived physical and mental health over time. Patients with cancer experience major stressful life events during the course of their disease, including loss of health and death. With therapeutic advancements, early-stage cancer, including DTC, is no longer incurable. However, psychosocial and spiritual factors, such as the uncertainty of cancer recurrence, changes in professional roles and family/social functioning, and demoralization, might also cause psychological distress and lead to a higher likelihood of depression [25,26]. For patients with DTC, their long-term quality of life is affected by the need to undergo numerous diagnostic and therapeutic procedures and the possible adverse effects or complications thereof [27]. A previous study reported a lower HRQoL after DTC surgery due to thyroid hormone dysregulation [10]. Almeida et al. reported that high-dose RAI therapy was the only clinical predictor of poor QoL [28]. Tagay et al. examined the relationship between depression and anxiety symptoms and HRQoL in a cross-sectional study of 136 patients with hypothyroidism who were on thyroid hormone withdrawal and were hospitalized for radioiodine administration. They reported that depression and age were independently associated with the physical health dimension, and that anxiety (62.5%) and depression (17.9%) were more prevalent in these patients than in the general population [29]. Consistent with this study, we observed that common psychological symptoms still predicted 1-month HRQoL impairment (mostly in physical component scores). Our study also indicated that the prevalence of clinical depressive disorders (TDQ ≥ 19) and CMDs (CHQ ≥ 5) in our baseline sample was 14% and 22%, respectively. A rare study on the psychological impact of DTC in Asian patients with DTC after surgery reported that 12.0% and 4.8% of patients had definite anxiety and depression, respectively, at a mean of 3.5 years after thyroid surgery [30]. These percentages were close to those in the general population and suggest that careful consideration is needed regarding whether DTC and surgery are the only causes of long-term anxiety or depression.

In the context of physical-related HRQoL in patients with DTC, the symptoms of postoperative hypothyroidism, including fatigue, weight gain, cramps, irritability, and memory impairment, have been demonstrated to result in a reduction in HRQoL [31]. Another study of 150 patients with DTC who were undergoing thyroid hormone withdrawal identified factors that correlated with HRQoL [32]. The majority of the most significant factors were physical in nature, including fatigue, intolerance to cold or heat, sleep disturbances, and weight gain. Consequently, it is essential for a HRQoL assessment to account for the physical symptoms patients have throughout the course of their thyroid cancer treatment, particularly during the process of thyroid hormone withdrawal. In a study conducted by Nygaard et al., the effectiveness of using rhTSH instead of thyroid hormone withdrawal in post-surgical evaluations was investigated [33]. The study employed a case-control design, comparing the outcomes of rhTSH administration and thyroid hormone withdrawal. The findings indicated that the rhTSH group exhibited superior results and a higher quality of life compared with the thyroid hormone withdrawal group. Similarly, our study revealed that patients who did not receive rhTSH had higher depression levels.

Our results also revealed that psychiatric follow-up and treatment history were significantly associated with improved physical components of HRQoL, such as PF and RP. As mentioned earlier, anxiety and depression may be independent risk factors for physical-related HRQoL impairments. This bidirectional psychosomatic relationship (mental health problems being both a risk factor and consequence of physical conditions) is well established. A classical study examined the relationship between psychiatric disorders and limitations in physical functioning in a sample of the US general population, showing that mood and anxiety disorders were independently associated with acute and chronic limitations in physical functioning [34]. In a cross-sectional study of 904 community-dwelling older adults, Garber et al. examined the physical and mental health-related correlates of physical functioning [35] and found that depression was associated with poorer physical functioning in addition to other physical-related factors. However, our results revealed that patients with DTC who had psychiatric disorders and psychiatric follow-up and treatment had improved physical functioning and fewer physical-related role limitations despite the evident impact of anxiety and depression on their HRQoL. This suggests that psychiatric follow-up and treatment may have beneficial effects on psychosomatic relationships. Other studies have reported similar results. A 2020 scoping review aimed to consolidate the evidence for modern depression treatment in people with cardiovascular disease [36] including 42 randomized controlled trials. The study concluded that selective serotonin reuptake inhibitors appear to be safe and have beneficial effects on depression (and some suggestions for cardiac benefits) in patients with coronary artery disease. Giusti et al. [37] performed a 5-year cohort study involving yearly psychological evaluations and demonstrated that regular evaluations of patients with DTC improved their HRQoL. The study had impressive findings regarding the beneficial effects on long-term quality of life of “only” an annual psychological evaluation. Their findings were similar to those of our 1-month study. Collectively, these data should serve as a reminder to healthcare providers regarding the importance of mental health in patients with DTC. Accordingly, a long-term follow-up study, extending over a period of 12 to 24 months, would be required to assess the long-term changes in psychological symptoms and HRQoL. Further structured and controlled clinical trials and analyses (with a particular focus on the effectiveness of supportive care and psychosocial interventions) are required to elucidate this psychosomatic interaction in patients with DTC. Furthermore, objective physical assessments are also necessary in the future, but not solely based on the patient’s perception of their own health status. These assessments should reflect the objective health status and medical conditions of the patient.

Our study had some limitations. First, the cohort of study participants was relatively limited in size, and the study was a single-center analysis that may not fully reflect the characteristics of the Chinese population. Furthermore, the number of patients with DTC in this cohort decreased marginally at the end of the study period; thus, the results may have been biased due to patient selection. However, the clinical course of most patients did not significantly progress during the 1-month follow-up period. Second, only 10% of the patients in our cohort received rhTSH, whereas most of the other patients underwent thyroid hormone withdrawal. Therefore, we excluded rhTSH as an independent variable from the regression models, precluding the determination of the interactive effects between exogenous or endogenous TSH stimulation, thyroid function (TSH and free T4 levels), and mental health relative to HRQoL in psychoendocrinology. Third, anxiety and depressive symptoms were obtained using a self-report questionnaire, with a lack of further clinical evaluation and diagnosis by psychiatrists or other mental health professionals. Finally, it should be noted that the relatively short follow-up period (one month post RAI) limits the ability to fully assess long-term mental health and HRQoL changes.

## 5. Conclusions

In conclusion, our results have corroborated those of previous cross-sectional studies and, on a longitudinal basis, have demonstrated the significance of psychological symptoms in relation to HRQoL. We identified that although anxiety and depressive symptoms may negatively affect certain HRQoL domains, psychiatric follow-up and treatment can improve the physical dimension. It is recommended that physicians involved in the follow-up of DTC patients devote particular attention to the assessment and management of patient depression and anxiety following radiotherapy ablation.

## Figures and Tables

**Table 1:** Clinicodemographic Characteristics of Participants (*n* = 200).

	*n* (%) or Mean (Standard Deviation)
Female Sex	148 (74.0)
Age	49.4 (12.7)
Marital Status	
Married	144 (72.0)
Single/Divorced/Widowed	56 (28.0)
Education	
0–6 years	34 (18.0)
7–12 years	104 (52.0)
>12 years	59 (30.0)
Hypertension	52 (26.0)
Diabetes	28 (14.0)
rhTSH use	20 (10.0)
Body Mass Index	25.8 (4.7)
CHQ ≥ 5	44 (22.0)
TDQ ≥ 19	28 (14.0)
Psychiatric Follow-up and Treatment History	22 (11.0)

Abbreviations: CHQ, Chinese Health Questionnaire; rhTSH, recombinant human thyroid-stimulating hormone; TDQ, Taiwanese Depression Questionnaire.

**Table 2:** Free T4 Levels and TDQ Scores of Patients With and Without rhTSH Use.

Variable ^a^	rhTSH Use		*p*-Value
	Yes	No	
	*n* = 20	*n* = 180	
Free T4 level (baseline)	2.1 (0.9)	0.3 (0.4)	<0.001
TDQ score (baseline)	6.0 (5.1)	9.5 (8.2)	0.01

^a^ mean (Standard Deviation). Abbreviations: free T4, free thyroxine; rhTSH, recombinant human thyroid-stimulating hormone; TDQ, Taiwanese Depression Questionnaire.

**Table 3:** Comparison of SF-36 Scores between our Cohort and the Taiwanese Reference Population.

SF-36 Scale	Subjects (*n* = 200)			Taiwan Reference Population *	
	Mean	Standard Deviation		Mean	Standard Deviation
Physical Functioning	83.43	17.48	≪	92.43	13.65
Role-Physical	59.60	40.36	≪	83.85	32.74
Bodily Pain	82.23	20.75	=	83.80	19.78
General Health	58.80	22.51	≪	66.60	21.26
Vitality	57.73	21.42	≪	68.19	18.96
Social Functioning	82.20	20.45	<	88.18	16.16
Role-Emotional	68.01	43.62	≪	82.36	34.00
Mental Health	70.02	18.96	<	74.09	17.57

* age: 45–54 years; <: *p* < 0.05; ≪: *p* < 0.001; =: not significant.

**Table 4:** Regression Coefficients of Factors Influencing HRQoL and Psychological Symptoms 1 Month after RAI Treatment.

	PF (β)	RP (β)	BP (β)	GH (β)	VT (β)	SF (β)	RE (β)	MH (β)	CHQ (β)	TDQ (β)
Age	−0.17	0.13	0.16	−0.25	0.11	−0.05	−0.20	−0.07	0.01	−0.03
Female	−2.08	−0.45	−1.85	4.19	0.27	3.27	7.85	2.22	0.90 *	2.36
BMI	0.27	0.67	0.33	0.47	0.20	0.21	−1.47	−0.26	0.04	0.08
Psychiatric follow-up history	11.60 *	33.21 *	−3.82	−5.31	0.46	7.94	14.81	−7.66	1.03	5.64 **
TSH	−0.001	0.03	0.02	−0.05	−0.02	0	−0.11	−0.04	0.001	0.01
Free T4	−0.84	1.01	−0.20	1.33	1.52	−3.11	−2.69	−0.27	−0.07	−1.10
CHQ	−2.33 *	−1.49	−3.54 **	−3.69 **	−0.36	−1.87	−2.07	−1.89	-	0.11
TDQ	−0.70	−2.82 ***	−0.60	−0.27	−0.93	−1.20 **	−1.73	−0.33	0.05	-
R^2^	0.347	0.306	0.3	0.323	0.231	0.342	0.292	0.427	0.433	0.412

* *p* < 0.05; ** *p* < 0.01; *** *p* < 0.001. Abbreviations: BMI, body mass index; BP, bodily pain; CHQ, Chinese Health Questionnaire; free T4, free thyroxine; GH, general health perceptions; MH, mental health; PF, Physical Functioning; RE, role limitations due to emotional problems; RP, role limitations due to physical problems; SF, social functioning; TDQ, Taiwanese Depression Questionnaire; TSH, thyroid-stimulating hormone; VT, vitality.

## Data Availability

The original contributions presented in the study are included in the article, further inquiries can be directed to the corresponding authors.
